# Dynamic observation and analysis of metabolic response to moxibustion stimulation on ethanol-induced gastric mucosal lesions (GML) rats

**DOI:** 10.1186/s13020-019-0266-5

**Published:** 2019-10-15

**Authors:** Yuan Zhang, Miao-sen Huang, Cai-chun Liu, Lin-yu Lian, Jia-cheng Shen, Qi-da He, Ying-jie Wang, Long-bin Zhang, Mi Liu, Zong-bao Yang

**Affiliations:** 1 Cancer Research Center, School of Medicine, Xiamen University, Xiamen, 361102 China; 2 College of Acupuncture and Moxibustion, Hunan University of Traditional Chinese Medicine, Changsha, 410208 China; 3 College of Acupuncture and Moxibustion, Fujian University of Traditional Chinese Medicine, Fuzhou, 350122 China

**Keywords:** Gastric mucosal lesion, Moxibustion, Metabolomics, ^1^H NMR, Dynamic analysis

## Abstract

**Background:**

Gastric mucosal lesion (GML) is the initiating pathological process in many refractory gastric diseases. And moxibustion is an increasingly popular alternative therapy that prevents and treats diseases. However, there are few published reports about developing pathology of GML and therapeutic mechanism of moxibustion treatment on GML. In this study, we investigated pathology of GML and therapeutic mechanism of moxibustion treatment on GML.

**Methods:**

The male Sprague-Dawley (SD) rats were induced by intragastric administration of 75% ethanol after fasting for 24 h and treated by moxibustion at Zusanli (ST36) and Liangmen (ST21) for 1 day, 4 days or 7 days. Then we applied ^1^H NMR-based metabolomics to dynamic analysis of metabolic profiles in biological samples (stomach, cerebral cortex and medulla). And the conventional histopathological examinations as well as metabolic pathways assays were also performed.

**Results:**

Moxibustion intervention showed a beneficial effect on GML by modulating comprehensive metabolic alterations caused by GML, including energy metabolism, membrane metabolism, cellular active and neurotransmitters function.

**Conclusions:**

Moxibustion can effectively treat gastric mucosal damage and effectively regulate the concentration of some related differential metabolites to maintain the stability of the metabolic pathway.

## Background

Gastric mucosal lesions (GML), characterized by gastric mucosal inflammation, erosion and bleeding, is commonly caused by emotional stress, physical and chemical factors, microorganisms and other factors [1]. It is widely known that GML is the initial pathological of refractory intractable gastric diseases such as Chronic Gastric Ulcers (CGU), chronic atrophic gastritis (CAG) and gastric cancer [2–4]. In modern medicine, GML is mainly treated by proton pump inhibitor (PPI) combined with antibiotics or histamine 2-antagonist, or surgery including helicobacter pylori eradication [5]. Although these treatments make a considerable contribution to control of GML, they are not very satisfying because of their limited application in clinical, higher expensive cost, long-term treatment as well as some adverse effects. Recently, the traditional Chinese medicine (TCM) has accepted by more and more people as a complementary alternative and increasingly accepted by the general people because of their significant efficacy, lower costs and few side-effects.

Moxibustion, as the intrinsic part of TCM, exerts therapeutic effects mainly by the heat stimulating acupoints from moxa burning [6, 7]. Currently, several studies have showed a therapeutic effective of moxibustion on GML [8–10]. And our previous studies have proved that moxibustion on acupoints of Stomach Meridian can effectively regulate the expression of genes or proteins associated with the proliferation and apoptosis signaling in gastric mucosa rats with GML [11, 12]. But the limited targets and single time point lead to a part of information for us to understanding pathology of GML and mechanism of moxibustion treatment since the complexity and unpredictable ways in organisms respond to stimuli due to diseases or interventions. So, the time-related metabolic profiles in organisms are urgently needed to observe for a more complete picture.

Metabolomics is an emerging system biological approach after genomics and proteomics. NMR-based metabolomics can be used to identify metabolic reactions and potential biomarkers, it can analyse hundreds or even thousands of samples in a relatively short period of time with the help of an auto sample [13]. Meanwhile, metabolomics is a powerful approach for studying pathophysiological processes, and metabolomic analysis of human samples may shed light on the mechanisms of gastric mucosal and help identify potential therapeutic targets [14]. It can analysis changes of metabolic profiles caused by disease and other stimuli from a holistic view, which coincides exactly with the TCM theory [15]. Nuclear magnetic resonance (NMR) is one of the most commonly used platform in metabolomics studies for its advantages including low per-sample cost, non-invasive and nonselective analysis [16]. Many studies have revealed that the metabolism of amino acids, sugars and lipids in the body is disordered after gastric mucosal injury. Metabolic abnormalities are one of the key pathological aspects of gastric mucosal injury [17, 18]. Therefore, the overall regulation characteristics of acupuncture and moxibustion are in line with the technical advantages of metabolomics. The use of metabolomics to study acupuncture theory and its mechanism of action may become the common language for Chinese traditional medicine to become international. Currently, metabolomics was increasingly applied for therapeutic mechanisms of TCM including moxibustion [19, 20]. In this study, to a better understanding for therapeutic mechanism of moxibustion on GML rats, the time-related metabolic profiles of samples (stomach, cerebral cortex and medulla) in GML rats were analyzed by ^1^H NMR-based metabolomics.

## Methods

### Reagents and materials

Deuterium oxide (D_2_O) and sodium 3-trimethylsilyl-(2, 2, 3, 3-d4)-1-propionate (TSP) were purchased from Sigma-Aldrich (St, Louis, MI, USA). Trichloroacetaldehyde hydrate was purchased from Sinopharm Chemical Reagent Co., Ltd (Shanghai, China). Hematoxylin solution and eosin solution for histological stain were purchased from Beijing Leagene Biotechnology Co., Ltd (Beijing, China). Moxa cones were obtained from Nanyang Xian Cao Pharmaceuticals Co., Ltd (Nanyang, China). All other used chemicals were of analytical grade.

### Animals

72 healthy male Sprague-Dawley (SD) rats (180–220 g) were used in this study. Animals were purchased from the experimental animal center of Wu (Permit Number: SCXK160803004). Animal care and experimental procedures used in this study were approved by the Animal Care and Use Committee of Xiamen University. The study was performed with the guidelines of National Institutes of Health for the Care and Use of Laboratory Animals. And the animals were euthanized after intervention.

### Animal preparation and grouping

All animals were maintained in a controlled condition (22 ± 1 °C, relative humidity of 65 ± 5%, and 12-h light/dark cycle) with ad libitum to feed and water. After 1-week adaptation, all rats were randomly separated into four groups: (a) control group, (b) gastric mucosal lesions (GML) model group, (c) GML rats with moxibustion treatment on the stomach meridian of Foot-Yangming (SMFY) acupoints (MA group), and (d) GML rats with moxibustion on non-acupoints (MNA). All rats except for controls were randomly divided into three subgroups (n = 6, per subgroup). In the first subgroup, rats were executed at 1 day after treatment. As mentioned above, the rats from second and third subgroup was sacrificed at 4 days and 7 days, respectively. All rats except for controls were established GML modeling with alcohol given by gavage with 75% ethanol solution at 4 mL/kg after deprived of food for 24 h [21].

### Moxibustion treatment

After GML modeling, Liangmen (ST 21) and Zusanli (ST 36) acupoints of rats in first, second and third subgroups were selected for MA stimulation for 1 day, 4 days and 7 days. And these two acupoints were located according to the Veterinary Acupuncture of China and Government Channel and Points Standard GB12346-90 of China. Corresponding non-acupoints were located 5 mm away from acupoints mentioned above. And these non-acupoints were neither related to stomach meridian nor overlap with other known acupoints.

For moxibustion treatment, moxa cones (height: 5 mm, diameter: 5 mm, “Han Medicine”, Nanyang, China) were ignited and fixed at 2 cm above the selected spots (a 30-min session per day) in MA group as well as the non-acupoints in MNA group. Rats in GML model group were performed with no treatment but similar administration.

### Biological sample collections

The animals from three subgroups were respectively sacrificed anesthetized with isoflurane in day 1, day 4 and day 7. Then the cerebral cortex, medulla and stomach tissue samples were excised and snap-frozen in liquid nitrogen immediately for tissue extraction. All of samples were stored at − 80 °C prior to analysis.

### Histopathological examination

After dehydration of gastric tissues samples fixed in 4% paraformaldehyde, routine paraffin imbedding was applied. Then the 5-μm thick sections were sliced. Deparaffinage and hydration of the sections were achieve by soaking in xylenes I, II and gradient alcohol solution (absolute alcohol, 95% alcohol, 85% alcohol and 75% alcohol). Subsequently, samples were washed in water and stained with hematoxylin and eosin. Finally, a histopathological examination was conducted under light microscopy.

### Evaluation of gastric ulcer index

The rats were sacrificed anesthetized by inhaled isoflurane after treatment. The stomach was cut open along the greater curvature and washed with 0.9% Nacl solution. The area of gastric ulcer was measured with a vernier caliper and the index of gastric ulcer was counted. The severity of gastric lesions are scored as follows: petechial spots = 1, erosions < 1 mm = 2, erosions between 1 and 2 mm = 3, erosions between 2 and 4 mm = 4, and erosions greater than 4 mm = 5, and when the width of ulceration was < 1 mm the score was multiplied by 2. Then, the final total lesion score was obtained from the summery of the partial scores.

### Biological samples preparation and ^1^H NMR experiments

According to our preliminary study [22], each of pre-weighed stomach, cerebral cortex, medulla samples (200 mg) were homogenated in 600 mL of CH_3_OH and 300 mL of H_2_O and then vortexed for 1 min. After partitioning on ice for 10 min, the samples were centrifuged for 10 min (10,000 rpm, 4 °C). Next, the upper supernatant were piped into tubes for lyophilization and then mixed with 600 μL D_2_O comprising sodium 3-trimethylsilyl-(2, 2, 3, 3-d4)-1-propionate (TSP). Finally, the mixture was transferred into 5 mm NMR tube to perform NMR analysis.

^1^H NMR spectra of these samples were captured using a Bruker 600 MHz spectrometer at 298 K as well. Standard 1D ^1^H spectra were obtained with a ‘NOESY’ (RD-901-t1-90°-tm-90°-acquire) pulse sequence. For each sample, 64 FIDs were collected into 64 K data points over a spectral width of 12,000 Hz with a relaxation delay of 6.5 μs. The metabolites in all samples of ^1^H NMR spectra were assigned according to reference in published literature [22–24], in-house developed NMR database and HMDB database.

### Data preprocessing and multivariate statistical analysis

MestReNova v9.0.1 software (Mestrelab Research, Santiago de Compostela, Spain) was utilized to phase and baseline correct the ^1^H NMR spectra from all samples. And the ^1^H NMR spectra were referenced to a single peak from TSP at 0.0 ppm and peak-aligned to deal with peak-shift problem. To obviate the influence of water, the chemical shift range of δ 4.70–5.2 ppm were removed. Then the spectra were segmented at δ 0.01 intervals across the region of 0.5–9.0 ppm. The integral values were normalized to a sum of all integrals in each spectrum to compensate for concentration differences between the samples. Thus, the forming date matrices were conducted further for multivariate analysis.

The ^1^H NMR spectral data was processed with SIMCA-P14.1 (Umetrics, Sweden) to elucidate patterns of metabolic profiles. Prior to multivariate analysis, the Pareto-scaling was conducted to decrease the effect of noise and artefacts in the models. First, a principal component analysis (PCA) was carried out for a natural separation among the all groups via visual inspection of the score plots in this study. Next, the projection to latent structures discriminant analysis (PLS-DA) and supervised orthogonal projection to latent structures discriminant analysis (OPLS-DA) were conducted to distinguish different groups. In addition, the quality of OPLS-DA model was assessed by parameter R2 for model fitness and parameter Q2 for model predictive ability. Subsequently, the corresponding S-plot of OPLS-DA model was carried out to discover the potential variables for differentiation. The potential biomarkers were filtered according to the variable importance in the project (VIP) of the established OPLS-DA model (VIP > 1.00) and an independent-sample t-test (*p* < 0.05) using (SPSS21.0).

## Results

### Histological morphology examinations

**Fig. 1:**
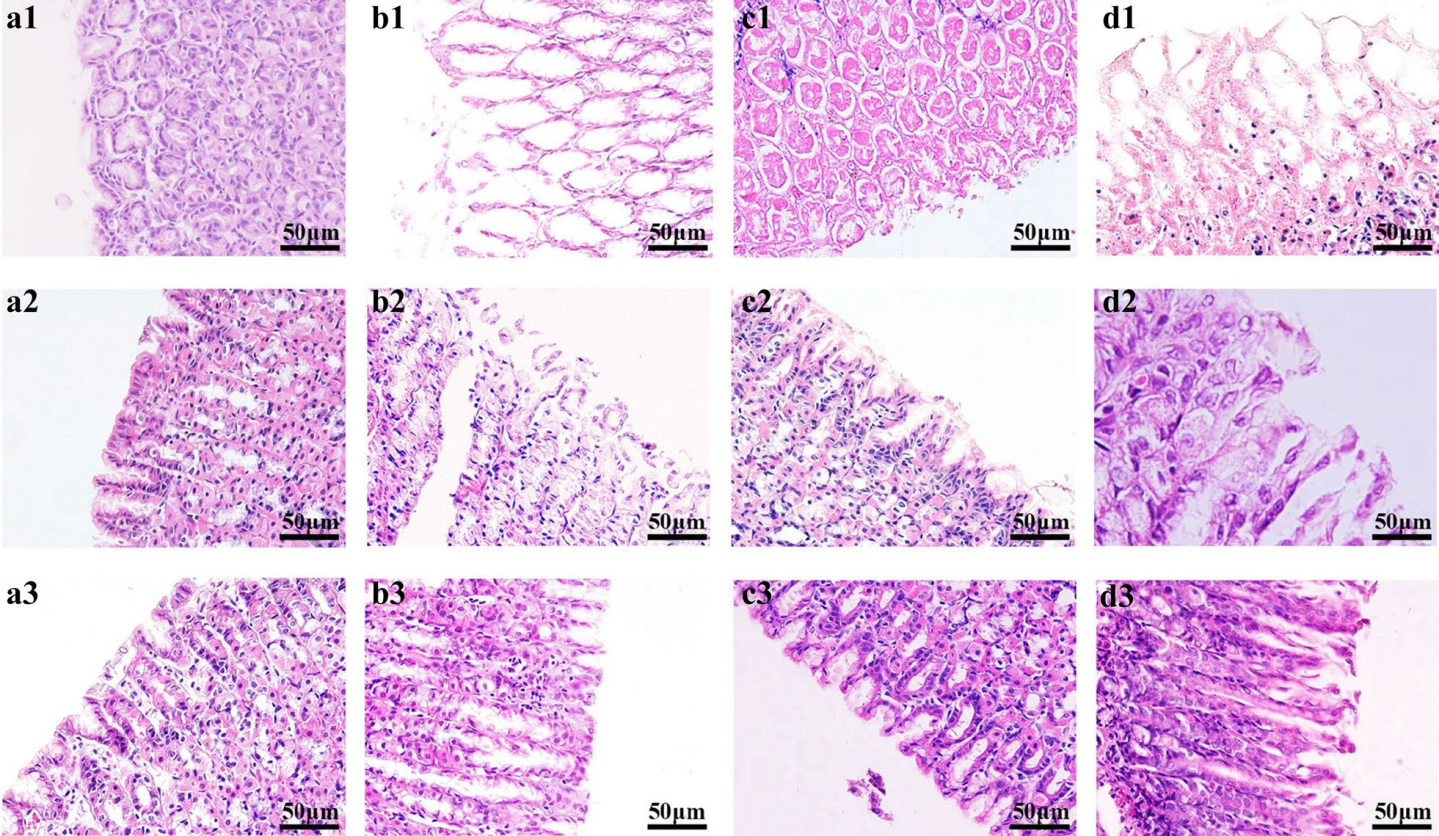
Histological morphology of gastric mucosa from 12 groups. **a1**, **b1**, **c1** and **d1**, rats in control group, gastric mucosal lesions model group, moxibustion-acupoint group and moxibustion-nonacupoint group at 1 day, respectively; **a2**, **b2**, **c2** and **d2**, rats in control group, gastric mucosal lesions model group, moxibustion-acupoint group and moxibustion-nonacupoint group at 4 days, respectively; **a3**, **b3**, **c3** and **d3**, rats in control group, gastric mucosal lesions model group, moxibustion-acupoint group and moxibustion-nonacupoint group at 7 days, respectively; Scale bars represent 50 μm in each group

Histological morphology examinations of the gastric mucosal tissues from rats in 12 groups were respectively conducted (Fig. 1 and Additional file 1: Figure S1). For controls in three time points, it was showed that the structure of mucous epithelium were preserved integrated with well-arranged cells and there were no inflammatory cellular infiltration in the submucosa and muscularis. At 1 day, rats except for the controls presented a seriously damaged structure of mucous membrane with a large number of cell necrosis and abscission, significant inflammatory cellular infiltration in submucosa and muscularis, where cells were arrayed in disordered state. The result was in accord with the gastric lesions indexes of rats in GML group, MA group and MNA group (Table 1), which were all higher significantly than the control at day 1 (all *p *< 0.01). Based on these observations, the rat modeling of GML was a successful replication. For the GML rats at 4 days and 7 days, the histological morphology examinations showed a partly improved in structure of mucosa and inflammatory cellular infiltration, indicating a self-restoring occurring during the development of GML. On the other hand, the gastric mucosa of GML rats with moxibustion treatment were decreased in lesions index at 4 days and improved in different degrees with the structure of gastric mucosa and inflammatory cellular infiltration at 4 days and 7 days, where ameliorated better than GML group at the same time point. These observations illustrate that moxibustion treatment a good effect on rats with GML rats. Besides, compared with the same time point of rats in MA group and MNA group, there is more significant improvement in gastric lesions index and gastric mucosa of GML rats with moxibustion on acupoints treatment. It suggests that moxibustion treatment on acupoints had relatively better effect on rats with GML.

**Table 1:** Gastric lesions indexes of rats in the four groups at day 1, 4 and 7 (mean ± S.D)

Group	Day 1 (*n *= 6)	Day 4 (*n *= 6)	Day 7 (*n *= 6)
Control	0.17 ± 0.41	0.67 ± 0.82	0.67 ± 0.82
GML	22.17 ± 2.56**	10.33 ± 2.80**	1.83 ± 1.94
MA	19.17 ± 2.56**	3.67 ± 2.50*^✦▲▲^	1.17 ± 1.1
MNA	20.17 ± 2.32**	6.83 ± 1.94**^▲^	1.67 ± 1.62

Data presented as mean ± standard error of the mean

* or **Denotes a statistical significance *p *< 0.05 or *p *< 0.01 when compared with the control group

^▲^ or ^▲▲^Denotes a statistical significance *p *< 0.05 or *p *< 0.01 when compared with the GML group

^✦^Denotes a statistical significance *p *< 0.05 when compared with the MNA group

### Gastric ulcer index detection

The gastric ulcer index was measured in 12 groups of rats (Table 1). Compare to the control, the gastric lesions indexes of GML group, MA group and MNA group were higher significantly at day 1 (all *p *< 0.01), indicating the GML modeling was replicated successfully. In addition, the gastric lesions index of rats in MA group was the lowest among the GML group, MA group and MNA group at day 4, demonstrating that moxibustion acupoints has better beneficial effective on gastric lesion in GML rats.

### ^1^H NMR profiles of stomach and brain tissues from rats

**Fig. 2:**
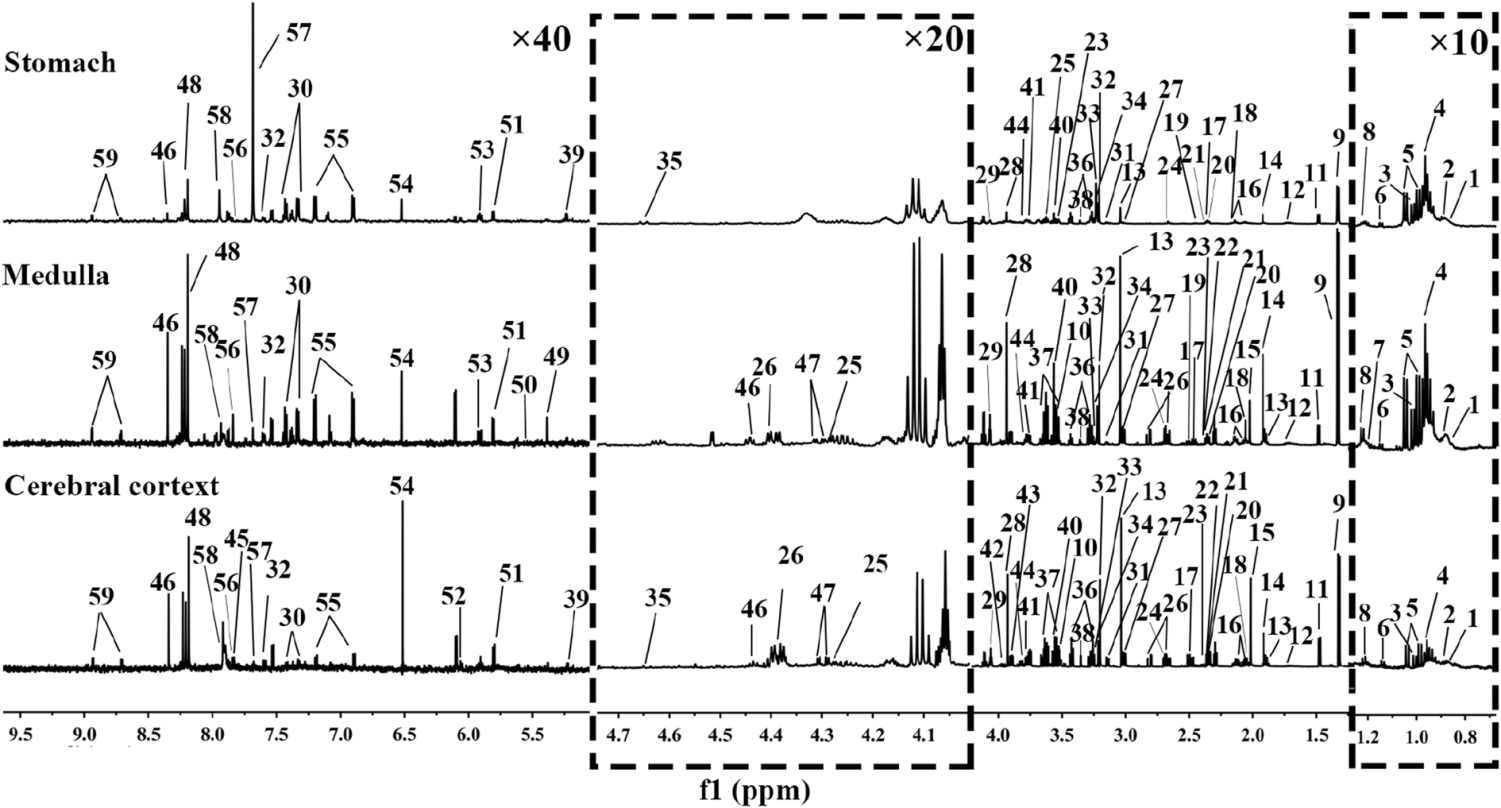
Typical ^1^H NMR spectra of aqueous extracts from stomach, medulla and cerebral cortex tissues. 1, Low density lipoprotein; 2, very low density lipoprotein; 3, isoleucine; 4, leucine; 5, valine; 6, 2-oxoisovalerate; 7, ethanol; 8, methylmalonate; 9, lactate; 10, threonine, 11, alanine; 12, lysine, 13, γ-aminobutyrate; 14, acetate; 15, *N*-acetyl aspartate; 16, glutamate; 17, glutamine; 18, methionine; 19, glutathione; 20, pyruvate; 21, oxaloacetate, 22, succinate, 23, α-ketoglutarate; 24, citrate, 25, sarcosine, 26, aspartate; 27, *N,N*-dimethylglycine; 28, creatine; 29, creatinine, 30, phenylalanine; 31, ethanolamine; 32, choline; 33, phosphocholine; 34, glycerophosphocholine; 35, β-glucose; 36, taurine; 37, myo-inositol; 38, methanol; 39, α-glucose; 40, glycine; 41, glycerol; 42, glycogen; 43, guanidoacetate; 44, serine; 45, hippurate; 46, inosine; 47, adenosine; 48, NAD+; 49, allantoin; 50, uridine diphosphate glucose; 51, uracil; 52, cytidine; 53, uridine; 54, fumarate; 55, tyrosine; 56, histidine; 57, 3-methylhistidine; 58, xanthine; 59, nicotinamide

To investigate the time-relative metabolic changes correlated with the therapeutic mechanism of moxibustion on GML, the metabolic profiles of stomach, cerebral cortex and medulla exacts were detected with ^1^H NMR-based metabolomics at 1 day, 4 days and 7 days. The main metabolites from the spectra were identified according to our own developed NMR database, Human Metabolome Database (HMDB: http://www.hmdb.ca/) and literature data. A list of quantified metabolites from different matrices is shown in Additional file 1: Table S1 and typical ^1^H NMR spectra of stomach, cerebral cortex and medulla extracts are demonstrated (Fig. 2).

**Fig. 3:**
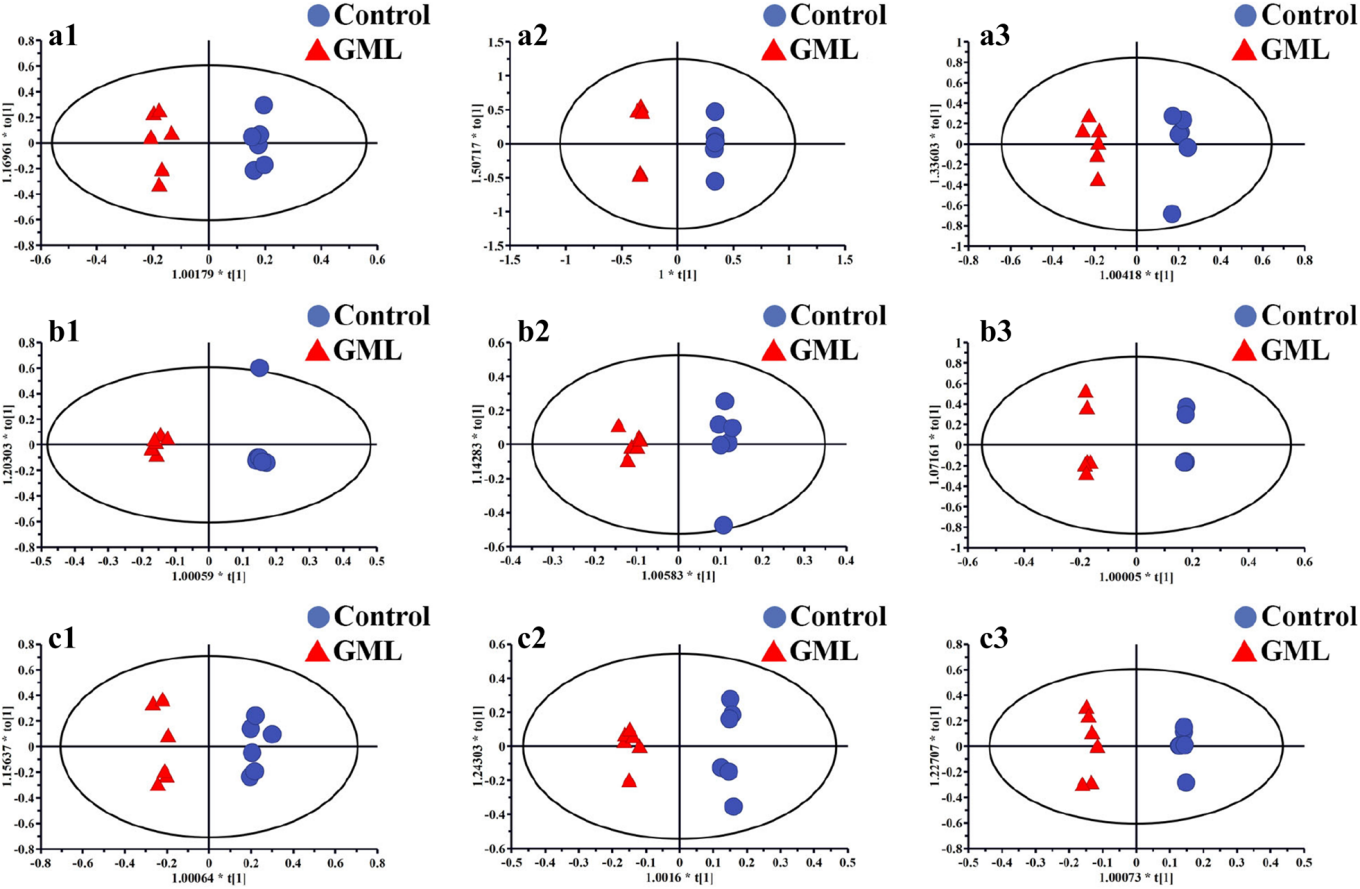
OPLS-DA scores plots from the GML rats and the control rats in stomach tissues at 1 day (**a1**, R2X = 0.695, R2Y = 0.987, Q2 (cum) = 0.68), 4 days (**a2**, R2X = 0.944, R2Y = 0.999, Q2 (cum) = 0.877) and 7 days (**a3**, R2X = 0.687, R2Y = 0.981, Q2 (cum) = 0.4); in cerebral cortex tissues at 1 day (**b1**, R2X = 0.743, R2Y = 0.993, Q2 (cum) = 0.937), 4 days (**b2**, R2X = 0.77, R2Y = 0.983, Q2 (cum) = 0.801) and 7 days (**b3**, R2X = 0.852, R2Y = 0.999, Q2 (cum) = 0.981); in medulla tissues at 1 day (**c1**, R2X = 0.821, R2Y = 0.981, Q2 (cum) = 0.82), 4 days (**c2**, R2X = 0.718, R2Y = 0.991, Q2 (cum) = 0.815) and 7 days (**c3**, R2X = 0.817, R2Y = 0.994, Q2 (cum) = 0.79)

**Fig. 4:**
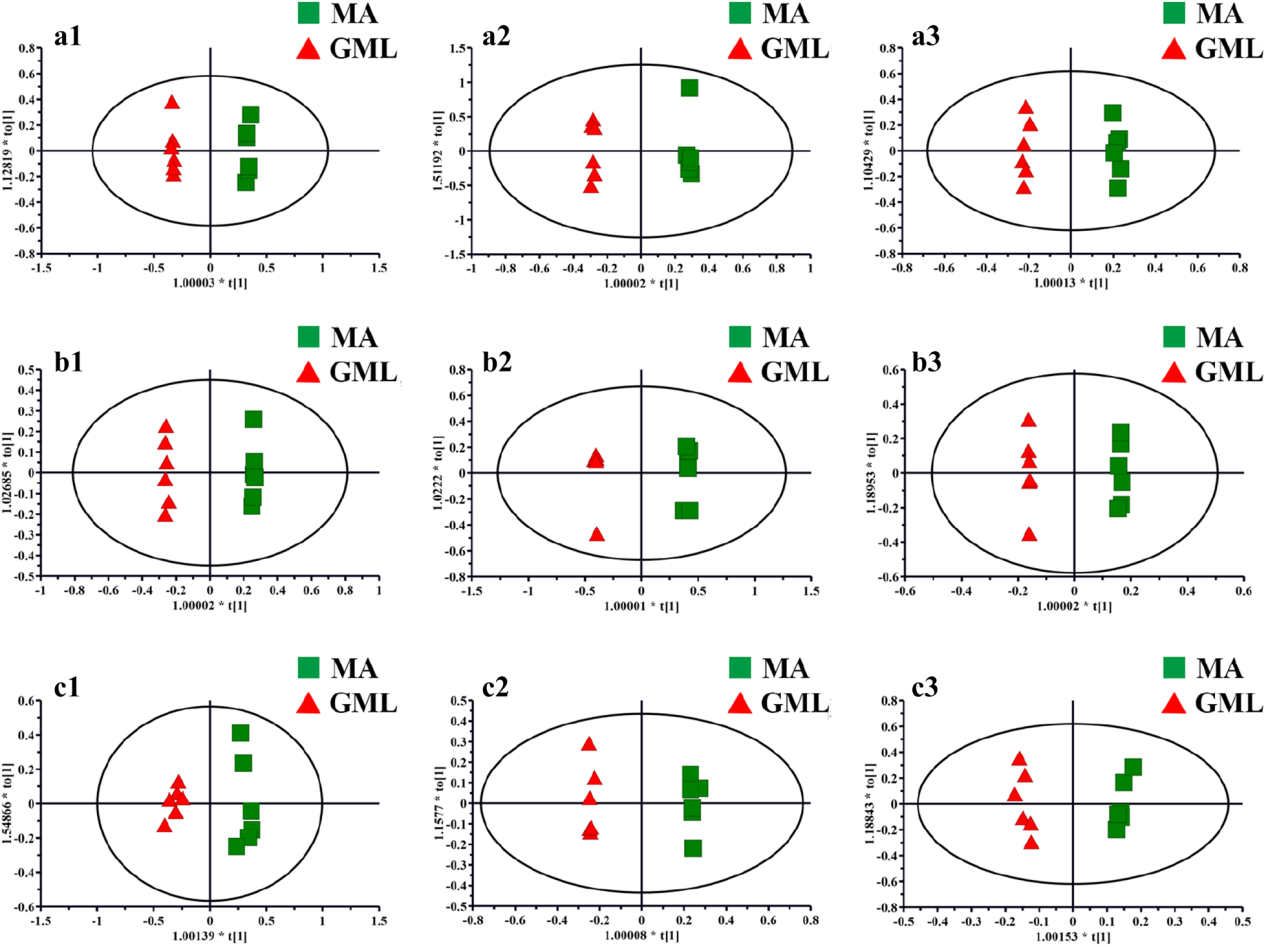
OPLS-DA scores plots from the GML rats and the GML rats with moxibustion acupoints (MA) treatment in stomach tissues at 1 day (**a1**, R2X = 0.729, R2Y = 0.999, Q2 (cum) = 0.959), 4 days (**a2**, R2X = 0.937, R2Y = 0.999, Q2 (cum) = 0.971) and 7 days (**a3**, R2X = 0.674, R2Y = 0.997, Q2 (cum) = 0.842); in cerebral cortex tissues at 1 day (**b1**, R2X = 0.759, R2Y = 0.999, Q2 (cum) = 0.996), 4 days (**b2**, R2X = 0.817, R2Y = 0.999, Q2 (cum) = 0.993) and 7 days (**b3**, R2X = 0.923, R2Y = 1, Q2 (cum) = 0.937); in medulla tissues at 1 day (**c1**, R2X = 0.739, R2Y = 0.974, Q2 (cum) = 0.791), 4 days (**c2**, R2X = 0.78, R2Y = 0.998, Q2 (cum) = 0.956) and 7 days (**c3**, R2X = 0.673, R2Y = 0.987, Q2 (cum) = 0.746)

Due to the complexity of the spectra, visual inspecting of these ^1^H NMR spectra showed less obvious difference. Therefore, all datasets were subsequently analyzed using PLS-DA and tow-group of OPLS-DA to discover any potential variables related to GML and moxibustion treatment. For all tissue types, the OPLS-DA scores from the control group and GML group showed obvious separations in three time points (Fig. 3), indicating a noticeable metabolic profiles perturbation occurred in GML. On the other hand, MA group separated obviously from GML group (Fig. 4), suggesting that moxibustion treatment had an obvious effect on GML.

In order to further filter the metabolites related to GML pathology and moxibustion treatment, we performed the corresponding S-plot and t-test (Additional file 1: Figures S2, S3).

Compared with the controls, the levels of β-glucose, α-glucose, pyruvate, oxaloacetate, citrate, acetate, isoleucine, leucine, valine, aspartate, phenylalanine, tyrosine and glutamate in stomach tissues of GML rats underwent significant changes as follows (Additional file 1: Table S2): (a) glutamine was decreased at 1 day; (b) pyruvate, oxaloacetate, glutamate were decreased at 1 day and 4 days; (c) β-glucose and α-glucose was found to increase at 1 day while decrease at 4 days; (d) acetate, isoleucine, leucine, valine, lysine, tyrosine and phenylalanine was decrease at 1 day while increase at 4 days; (e) citrate and aspartate were increase at 7 days. And the metabolites mentioned above in rats with moxibustion treatment, compared with GML rats, were reversed as follows: (a) glutamine, β-glucose, acetate, isoleucine, leucine, valine, lysine, tyrosine and phenylalanine were reversed by both moxibustion on acupoints and non-acupoints for 1 day; (b) aspartate was up-regulation by both moxibustion acupoints and non-acupoints for 1 day; (c) α-glucose, pyruvate, oxaloacetate and glutamate were reversed by moxibustion on acupoints for 1 day while by moxibustion non-acupoints for 4 days; (d) citrate was increased by moxibustion on acupoints for 1 day while by moxibustion non-acupoints for 4 days.

On the other hand, for metabolites in cerebral cortex tissues of GML rats, the levels of creatine, succinate, α-ketoglutarate, fumarate, GPC, taurine, glutamate and glutamine were altered compared with the controls as follows (Additional file 1: Table S3): (a) creatine was increased at 1 day; (b) succinate, α-ketoglutarate, fumarate and taurine were decreased at 1 day; (c) GPC was increased at 1 day and decreased at 7 days; (d) glutamate and glutamine were decreased at 1 day and at 4 days while increased at 7 days. And compared with GML groups, the levels of (a) succinate, creatine, glycerophosphocholine, fumarate were reversed by moxibustion acupoints at 1 day and succinate showed an increasing trend at 7 days while creatine and glycerophosphocholine showed a descended trend at 4 days by both moxibustion acupoints and non-acupoints; (b) taurine was elevated by both moxibustion acupoints and non-acupoints for 1 day and keep increasing at 4 days by moxibustion acupoints; (c) glutamate and glutamine were elevated at 1 day and 4 days by both moxibustion acupoints and non-acupoints and glutamate showed a declined trend to normal at 7 days by moxibustion acupoints.

Additionally, we also found that, in medullary samples of rats, the levels of citrate, lactate, phenylalanine, glutathione, glycine, serine and methionine were significantly changed by GML modeling (Additional file 1: Table S4): (a) lactate was increased at 1 day; (b) citrate was decreased at 1 day; (c) glycine and phenylalanine were decreased at 1 day and 4 days; (d) methionine and serine were decreased at 1 day while increased at 4 days and 7 days; (e) glutathione was decreased at 1 day and increased at 7 days. And these metabolites were reversed in response to moxibustion treatments for 1 day. Threonine, citrate and glutathione kept increasing significantly at 4 days by both moxibustion acupoints and non-acupoints treatments. Specially, the levels of serine at 4 days and phenylalanine at 7 days kept a noticeable increasing by moxibustion acupoints.

## Discussion

It is known that the functional dyspepsia is closely related to brain-gut peptide (BGP), which is effective in regulating various physiological functions in gastrointestinal tract by both enteric nervous system and central nervous system [25]. Interestingly, it has been reported that the gut-brain axis plays a potential role in gastric damage and protection [26]. And the cerebral cortex and medulla as the main tissue of the brain-intestinal axis, the effects of the brain-intestinal axis on the gastrointestinal tract can be reflected by the changes of the cerebral cortex and medulla in the whole experimental process. Meanwhile, in Traditional Chinese Medicine (TCM), the stomach meridian had an important connection with the stomach. The stomach diseases can be treated by stimulating the acupoints which on the stomach meridian [11, 27]. So the stomach is mainly related to meridians and can visually reflect local changes. The development of gastric mucosa lesions is a complex process including the occurrence and healing [28]. For all sample types, the different levels of changes caused by GML modeling and reversed by moxibustion treatment may bring about characteristic metabolomics profiles of GML modeling and moxibustion treatment, which will be discussed in further detail as following (Additional file 1: Figure S4).

### Metabolic alterations in stomach tissues

**Fig. 5:**
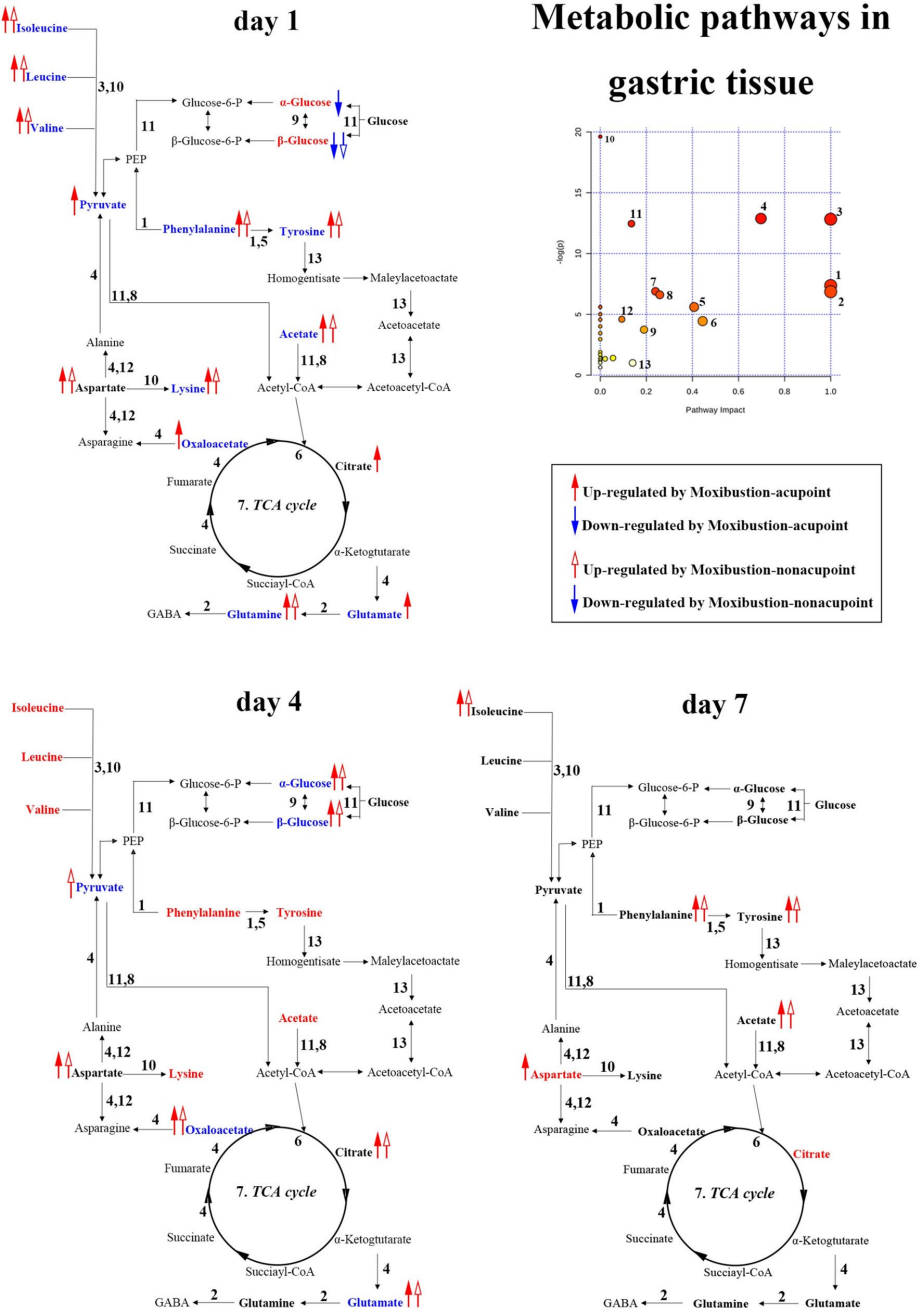
Disturbed metabolic pathways in stomach related to GML pathology and moxibustion treatment at three time points. 1, Phenylalanine, tyrosine and tryptophan biosynthesis; 2, d-glutamine and d-glutamate metabolism; 3, valine, leucine and isoleucine biosynthesis; 4, alanine, aspartate and glutamate metabolism; 5, phenylalanine metabolism; 6, glyoxylate and dicarboxylate metabolism; 7, citrate cycle (TCA cycle); 8, pyruvate metabolism; 9, starch and sucrose metabolism; 10, Aminoacyl-tRNA biosynthesis; 11, glycolysis or gluconeogenesis; 12, arginine and proline metabolism; 13, tyrosine metabolism. Metabolites in bold were remarked metabolites associated with moxibustion treatment on GML rats. (Those in red (or blue) indicate concentration increases (or decreases) in comparison with the control group, while ones in black present no insignificant change)

Glucose is the primary source of energy involving in glycolysis or gluconeogenesis. And pyruvate is a key intermediate in metabolism of carbohydrates, fats and proteins [29, 30]. Additionally, acetate is mainly utilized in the form of acetyl-CoA, an essential metabolite in three carboxylic acid (TCA) cycle. Besides, citrate and oxaloacetate are also important intermediates during TCA cycle. Previously, decreased citrate in stomach extracts was also recognized as potential biomarker for severe gastric ulcers [23]. In current study, alteration of glucose caused by GML at 1 day and 4 days indicates that GML modeling may bring an unbalance between the glycolysis and gluconeogenesis. In addition, lower levels of pyruvate, acetate, citrate and oxaloacetate in stomach tissues of GML rats at 1 day while increased concentration of citrate at 7 days suggest that GML modeling may cause an inhibition in TCA cycle for energy releasing and this inhibition disappears with the improvement of lesions. On the other hand, compared with moxibustion non-acupoints treatment, the more levels of metabolites mentioned above can be revered after moxibustion acupoints treatment for 1 day, suggesting that moxibustion acupoints treatment may play an important role in regulating energy metabolism at early stage during development of GML (Fig. 5).

Leucine, isoleucine and valines, branched-chain amino acids (BCAAs), play a functional role in accelerating mitochondrial metabolism, which lead to promote cell metabolism and improve immune-related function and gastrointestinal health [31–35]. And lysine is a precursor of cadaverine, an essential factor in fatty acid metabolism through transporting fat into the mitochondria for subsequent β-oxidation [36]. Phenylalanine is required for the synthesis of tyrosine, which readily passes the blood–brain barrier and is a precursor for the neurotransmitters dopamine, norepinephrine and epinephrine. Glutamate can interconvert into glutamine, which is vital energy source to promote mucosal and play important role in maintaining the structure and function of intestinal mucosa [37]. Aspartate is another nonessential amino acid and plays beneficial roles in anti-oxidative capacity, immunity, gastrointestinal absorptive and mucosal barrier function [38, 39]. Depletion in these amino acids in stomach tissues of GML rats at 1 day indicates that GML modeling may lead to disorders in immune-and neurotransmitters-related function as well as anti-oxidative capacity. And this depletion is reversed with the improvement of lesions. On the other hand, most of these amino acids are reversed for 1 day and keep a increasing during the development process of lesions, indicating that moxibustion have a beneficial treatment by regulating anti-oxidative capacity, immunity and the function of neurotransmitters (Fig. 5).

### Metabolic alterations in brain

**Fig. 6:**
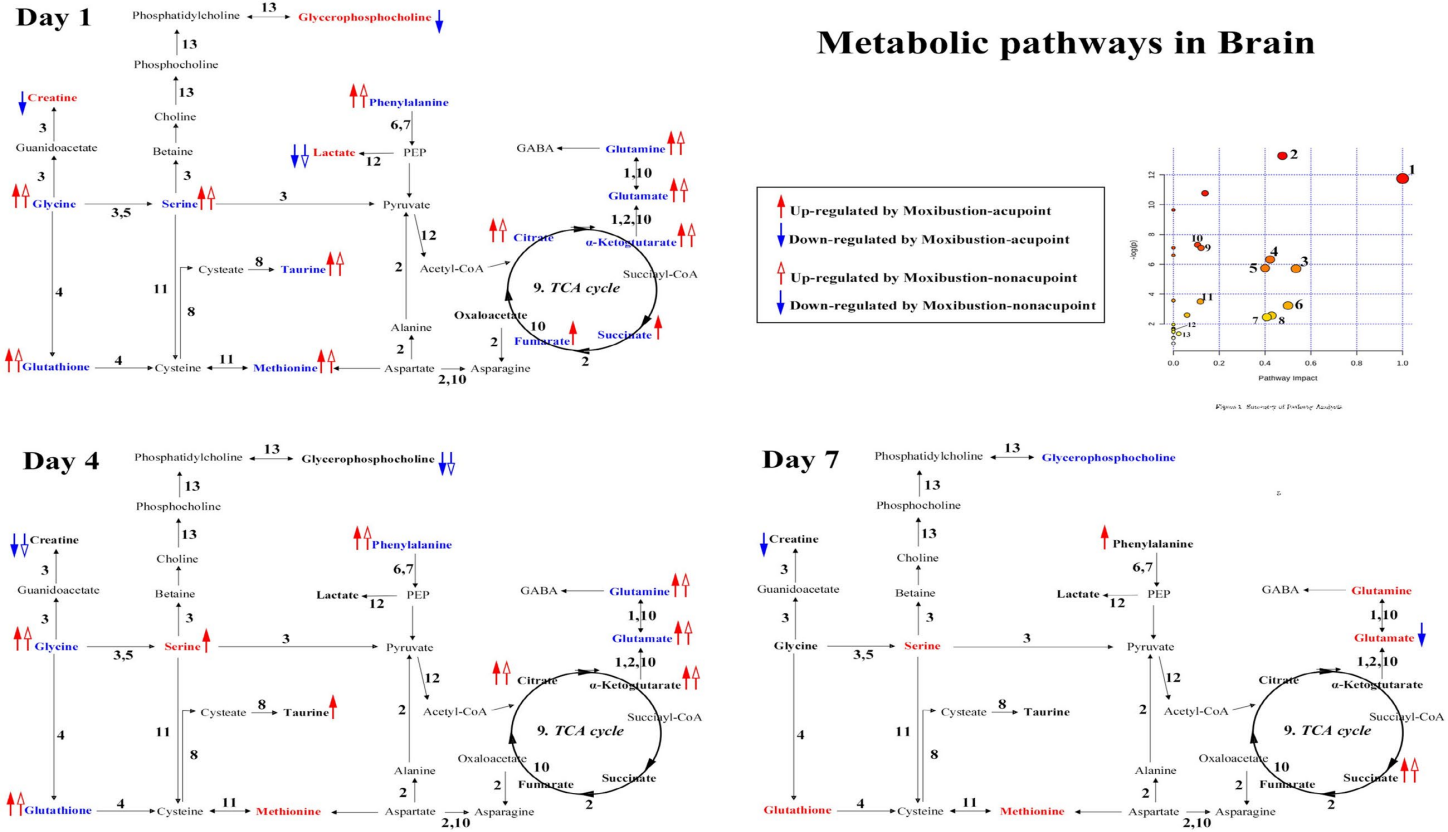
Disturbed metabolic pathways in brain related to GML pathology and moxibustion treatment at three time points. 1, d-Glutamine and d-glutamate metabolism; 2, alanine, aspartate and glutamate metabolism; 3, glycine, serine and threonine metabolism; 4, glutathione metabolism; 5, methane metabolism; 6, phenylalanine, tyrosine and tryptophan biosynthesis; 7, phenylalanine metabolism; 8, taurine and hypotaurine metabolism; 9, citrate cycle (TCA cycle); 10, arginine and proline metabolism; 11, cysteine and methionine metabolism; 12, pyruvate metabolism; 13, glycerophospholipid metabolism. Metabolites in bold were remarked metabolites associated with Moxibustion treatment on GML rats. [Those in red (or blue) indicate concentration increases (or decreases) in comparison with the control group, while ones in black present no insignificant change]

Citrate, α-ketoglutarate (α-KG), succinate and fumarate are important intermediates in TCA cycle. And lactate is final product from pyruvate in anaerobic cellular metabolism. Creatine functions as part of the cell’s energy shuttle that it can transfer ATP and provide energy through catalyzed to phosphocreatine and ADP [22]. Alteration of these metabolites in brain of GML rats suggests again that deficiency energy is an obvious pathology of GML. And the reversion by moxibustion acupoints treatment after 1 day, only citrate, α-KG and lactate reversed by moxibustion non-acupoints, also indicates again that moxibustion acupoints treatment can improve energy metabolism in GML rats (Fig. 6).

Glutathione is vital coenzymes with the crucial thiol group that make it have an effective antioxidant and can be synthesized from cysteine [40]. Glycine and serine are important metabolic intermediates in glutathione metabolism. Taurine is synthesized from cysteine and has a vital role in antioxidation, neuroprotective, membrane stabilization and osmoregulation [41–43]. Thus, serine and glycine can be used to enhance anti-oxidative reactions, improve immunity and prevent tissue injury [44, 45]. Methionine is known as a donator of the sulfur atom to form cysteine. So lower levels of serine, glycine, glutathione and methionine in medulla extracts as well as taurine in cortex extracts of GML rats and the depletion is reversed by moxibustion acupoints for 1 day and keep a increasing during the development process of lesions for 4 days or 7 days (Fig. 6), suggesting that GML modeling may bring an weaken antioxidant ability and moxibustion treatment has good effect in improving them.

Besides, glycerophosphocholine (GPC) is formed in the breakdown of phosphatidylcholine (PC), a major component of lecithin contributing to cellular proliferation and apoptosis [46, 47]. Increased GPC in cortex of GML rats may associate to a damaged in cell membrane [22], while its self-recovery and even reversion to low levels after 7 days indicates an increased acquirement in synthesis of cell membranes to repair the previous damage (Fig. 6). But the levels of GPC were reversed by moxibustion acupoints treatment for 1 day suggesting that moxibustion acupoints treatment has an effective on accelerating the cellular active.

In addition, glutamine is a precursor for GABA and it is supposed to be linked with positive intestinal effects, including maintenance of gut barrier function, aiding intestinal cell proliferation and differentiation and ant oxidative stress [48, 49]. Phenylalanine is known as a precursor for neurotransmitters catecholamines including tyramine, dopamine, epinephrine and norepinephrine [50]. The down-regulation in levels of glutamate, glutamine and phenylalanine in brains of GML rats suggests a disturbance in neuronal signaling pathways caused by GML. While the reversion by moxibustion treatment gives an indication that moxibustion treatment has a good effect on GML by improving neurotransmitters function (Fig. 6).

## Conclusions

Together, these findings suggests GML modeling brought disorders in energy metabolism, antioxidant ability, cellular active and function of neurotransmitters in stomach and brain of rats. Although GML rats had some self-restoration abilities, the moxibustion treatment showed an obvious improvement effects on GML especially in regulating energy metabolism and cellular active. In addition, moxibustion treatment also has a beneficial effect on GML by regulating antioxidant ability and function of neurotransmitters. The current study will be beneficial for better understanding the time-relative therapeutic mechanism of moxibustion on GML.

## Supplementary information

**Additional file 1. Figure S1.** Histological morphology of gastric mucosa from 12 groups. (A1, B1, C1 and D1, rats in control group, gastric mucosal lesions model group, moxibustion-acupoint group and moxibustion-nonacupoint group at 1 day, respectively; A2, B2, C2 and D2, rats in control group, gastric mucosal lesions model group, moxibustion-acupoint group and moxibustion-nonacupoint group at 4 days, respectively; A3, B3, C3 and D3, rats in control group, gastric mucosal lesions model group, moxibustion-acupoint group and moxibustion-nonacupoint group at 7 days, respectively; Scale bars represent 200 μm in each group.) **Figure S2.** Corresponding S-plots from the GML rats and the control rats in stomach (a1, a2 and a3), cerebral cortex (b1, b2 and b3) and medulla (c1, c2 and c3) tissues for three time points. **Figure S3.** Corresponding S-plots from the GML rats and the GML rats with moxibustion acupoints(MA) treatment in stomach (a1, a2 and a3), cerebral cortex (b1, b2 and b3) and medulla (c1, c2 and c3) tissues for three time points. **Figure S4.** Summaries of metabolic pathways in gut-brain integration altered moxibustion treatment on GML rats. Moxibustion intervention showed beneficial effects by regulating many GML-induced metabolic changes involved in energy metabolism, amino acids metabolism and membrane metabolism. (1, Phenylalanine, tyrosine and tryptophan biosynthesis; 2, d-Glutamine and d-glutamate metabolism; 3, Valine, leucine and isoleucine biosynthesis; 4, Alanine, aspartate and glutamate metabolism; 5, Glycine, serine and threonine metabolism; 6, Glutathione metabolism; 7, Phenylalanine metabolism; 8, Methane metabolism; 9, Glyoxylate and dicarboxylate metabolism; 10, Taurine and hypotaurine metabolism; 11, Citrate cycle (TCA cycle); 12, Pyruvate metabolism; 13, Starch and sucrose metabolism; 14, Aminoacyl-tRNA biosynthesis; 15, Glycolysis or Gluconeogenesis; 16, Arginine and proline metabolism; 17, Cysteine and methionine metabolism; 18, Tyrosine metabolism; 19, Primary bile and biosynthesis; 20, Glycerophospholipid metabolism.). **Table S1.** Peak attribution of the main marked metabolites in 1H-NMR spectra of stomach, cerebral cortex, medulla. **Table S2.** The alteration metabolites from stomach tissue. **Table S3.** The alteration metabolites from cerebral cortex tissue. **Table S4.** The alteration metabolites from medulla tissue.

## Data Availability

The all original data supported the findings of this study were supplied by Zong-bao Yang under license and cannot be made freely available. Requests for access to these data should be made to Zongbao Yang, yangzb@xmu.edu.cn.

## Abbreviations

GML: gastric mucosal lesion
SD: Sprague–Dawley
ST36: Zusanli
ST21: Liangmen
CGU: Chronic Gastric Ulcers
CAG: chronic atrophic gastritis
PPI: proton pump inhibitor
TCM: the traditional Chinese medicine
NMR: nuclear magnetic resonance
D_2_O: deuterium oxide
TSP: sodium 3-trimethylsilyl-(2, 2, 3, 3-d4)-1-propionate
SMFY: Foot-Yangming
MNA: GML rats with moxibustion on non-acupoints
PCA: principal component analysis
PLS-DA: the projection to latent structures discriminant analysis
OPLS-DA: orthogonal projection to latent structures discriminant analysis
VIP: variable importance in the project
TCA: three carboxylic acid
BCAAs: branched-chain amino acids
α-K: Gα-ketoglutarate
GPC: glycerophosphocholine
PC: phosphatidylcholine
