# Supplementary material for: Dynamic observation and analysis of metabolic response to moxibustion stimulation on ethanol-induced gastric mucosal lesions (GML) rats
Source: Chin Med. 2019 Oct 15;14:44. doi: 10.1186/s13020-019-0266-5 (PMC6794790; doi:10.1186/s13020-019-0266-5)
Supplement: Supplementary file 1 — Additional file 1. Figure S1. Histological morphology of gastric mucosa from 12 groups. (A1, B1, C1 and D1, rats in control group, gastric mucosal lesions model group, moxibustion-acupoint group and moxibustion-nonacupoint group at 1 day, respectively; A2, B2, C2 and D2, rats in control group, gastric mucosal lesions model group, moxibustion-acupoint group and moxibustion-nonacupoint group at 4 days, respectively; A3, B3, C3 and D3, rats in control group, gastric mucosal lesions model group, moxibustion-acupoint group and moxibustion-nonacupoint group at 7 days, respectively; Scale bars represent 200 μm in each group.) Figure S2. Corresponding S-plots from the GML rats and the control rats in stomach (a1, a2 and a3), cerebral cortex (b1, b2 and b3) and medulla (c1, c2 and c3) tissues for three time points. Figure S3. Corresponding S-plots from the GML rats and the GML rats with moxibustion acupoints(MA) treatment in stomach (a1, a2 and a3), cerebral cortex (b1, b2 and b3) and medulla (c1, c2 and c3) tissues for three time points. Figure S4. Summaries of metabolic pathways in gut-brain integration altered moxibustion treatment on GML rats. Moxibustion intervention showed beneficial effects by regulating many GML-induced metabolic changes involved in energy metabolism, amino acids metabolism and membrane metabolism. (1, Phenylalanine, tyrosine and tryptophan biosynthesis; 2, d-Glutamine and d-glutamate metabolism; 3, Valine, leucine and isoleucine biosynthesis; 4, Alanine, aspartate and glutamate metabolism; 5, Glycine, serine and threonine metabolism; 6, Glutathione metabolism; 7, Phenylalanine metabolism; 8, Methane metabolism; 9, Glyoxylate and dicarboxylate metabolism; 10, Taurine and hypotaurine metabolism; 11, Citrate cycle (TCA cycle); 12, Pyruvate metabolism; 13, Starch and sucrose metabolism; 14, Aminoacyl-tRNA biosynthesis; 15, Glycolysis or Gluconeogenesis; 16, Arginine and proline metabolism; 17, Cysteine and methionine metabolism; 18, Tyrosine metabolism; [file 13020_2019_266_MOESM1_ESM.pdf]

Dynamic Observation and Analysis of Metabolic response to Moxibustion stimulation on ethanol-induced gastric mucosal lesions (GML) Rats

Yuan Zhang[1,2], Miao-sen Huang[†1,3], Cai-chun Liu[1], Lin-yu Lian[1,3], Jia-cheng Shen[1], Qi-da He[1,3],Ying-jie Wang[1], Long-bin Zhang[1], Mi Liu[2], Zong-bao Yang[1※]

[1] *[1]Cancer Research Center, School of Medicine, Xiamen University, Xiamen 361005, China*

[2]College of Acupuncture and Moxibustion, Hunan university of Traditional Chinese Medicine, Changsha 410208, China

[3]College of Acupuncture and Moxibustion, Fujian university of Traditional Chinese Medicine, Fuzhou 350122, China

※ *[1]Cancer Research Center, School of Medicine, Xiamen University, Xiamen 361005, China*

[†]Miao-sen Huang is the Co-first author

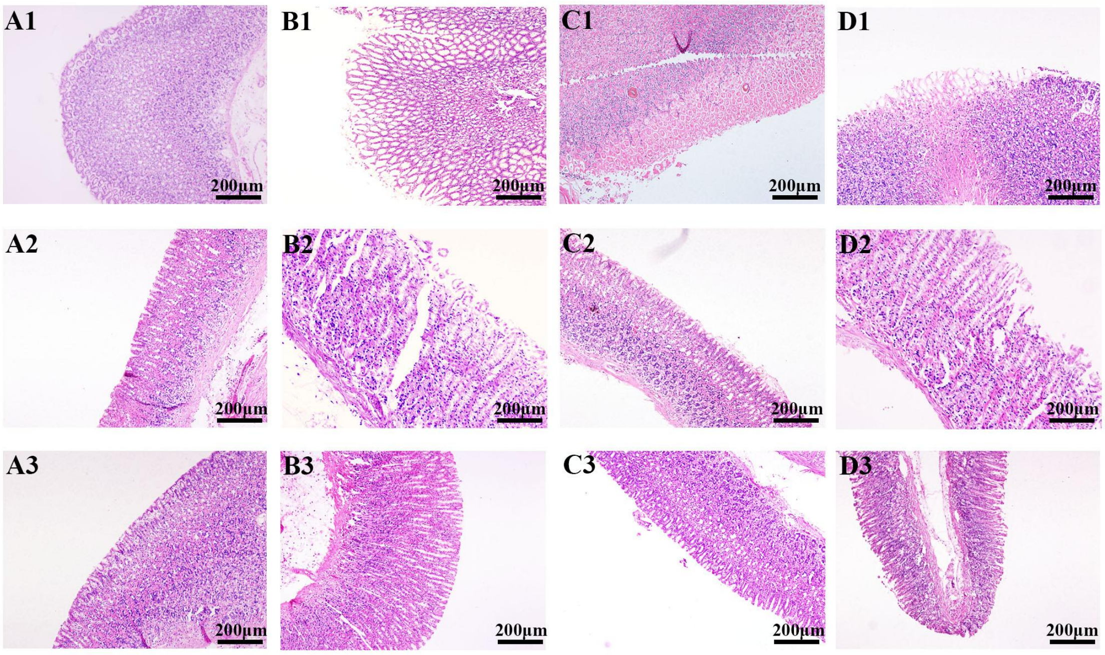

Figure. S1 Histological morphology of gastric mucosa from 12 groups. (A1, B1, C1 and D1, rats in control group, gastric mucosal lesions model group, moxibustion-acupoint group and moxibustion-nonacupoint group at 1day, respectively; A2, B2, C2 and D2, rats in control group, gastric mucosal lesions model group, moxibustion-acupoint group and moxibustion-nonacupoint group at 4days, respectively; A3, B3, C3 and D3, rats in control group, gastric mucosal lesions model group, moxibustion-acupoint group and moxibustion-nonacupoint group at 7days, respectively; Scale bars represent 200μm in each group.)

Table S1 Peak attribution of the main marked metabolites in ^{1}H-NMR spectra of stomach, cerebral cortex, medulla

NO.	Metabolites	δ^{1}H/ppm	Moieties	Sample
1	LDL	0.86(br)	CH3-(CH2)n-	St, M, CC
2	VLDL	0.89(br)	CH3-(CH2)n-	St, M, CC
3	Isoleucine	0.94(t); 1.01(d)	δ-CH_3; β-CH3	St, M, CC
4	Leucine	0.96(t); 1.70(m)	CH_3; CH_2&γ-CH	St, M, CC
5	Valine	0.99(d); 1.04(d)	γ-CH_3; γ-CH'_3	St, M, CC
6	OIV	1.12(d)	CH3	M, CC

7	Ethanol	1.19(t);3.66(q)	CH3; CH2	M
8	Methylmalonate	1.23(d)	CH_3	St, M, CC
9	Lactate	1.33(d); 4.11(q)	CH_3; CH	St, M, CC
10	Threonine	1.33(d);3.59(d);4.25(m)	CH3; α -CH; β -CH	M, CC
11	Alanine	1.48(d); 3.78(q)	CH_3; CH	St, M, CC
12	Lysine	1.73(m);1.88(m); 3.03(t)	γ-CH2; δ-CH2;β-CH2; ε-CH2; α-CH	St, M, CC
13	GABA	1.90(m);2.30(t); 3.02(t)	α-CH_2; β-CH_2	M, CC
14	Acetate	1.92(s)	CH_3	St, M, CC
15	NAA	2.02(s); 2.50(m)	CH_3	M, CC
16	Glutamate	2.05(m);2.13(m);2.35(m)	β-CH	St, M, CC
17	Glutamine	2.14(m);2.46(m)	β-CH_2	St, M, CC
18	Methionine	2.14(s);2.16(m);2.64(t); 3.86(t)	S-CH_3;β-CH_2;S-CH_2; α-CH	St, M, CC
19	Glutathione	2.17(m); 2.55(m)	β-CH_2; γ-CH_2	St, M
20	Pyruvate	2.37(s)	CH_3	St, M, CC
21	Oxaloacetate	2.38(s)	CH_2	St, M, CC
22	Succinate	2.41(s)	CH	M, CC
23	α-Ketoglutarate	2.45(t); 3.01(t);	γ-CH_2; β-CH_2	M, CC
24	Citrate	2.54(d); 2.69(d)	CH_2	St, M, CC
25	Sarcosine	2.74(s);3.60(s)	CH3; CH2	St
26	Aspartate	2.67(dd);2.82(dd);3.90(dd)	β-CH; β-CH'	St, M, CC
27	DMG	2.93(s); 3.73(s)	CH3; CH2	St, M, CC
28	Creatine	3.04(s); 3.93(s)	CH_3; CH_2	St, M, CC
29	Creatinine	3.05(s); 4.06(s)	CH_3; CH_2	St, CC
30	Phenylalanine	3.13(m);3.28(m);7.33(d);7.38(t)	β-CH´; o-CH; p-CH	St, M, CC
31	Ethanolamine	3.14(t); 3.84(t)	CH_2NH_2; CH_2OH	St, M, CC
32	Choline	3.20(s);3.52(m);4.07(m)	CH_3;N-CH_2;O-CH_2	St, M, CC
33	Phosphocholine	3.22(s);3.59(m);4.17(m)	CH_3;N-CH_2;O-CH_2	St, M, CC
34	GPC	3.23(s); 3.96(m)	CH_3; CH&O-CH_2	St, M, CC
35	β-Glucose	3.25(dd);3.51(t);3.46(m); 3.49(t);3.72(dd);3.90(dd); 4.65(d)	CH(2);CH(4);CH(5); CH(3);CH(6);CH(6’); CH(1)	St, CC
36	Taurine	3.27(t);3.43(t)	CH_3; α-CH; β-CH	St, M, CC
37	Myo-inositol	3.28(t);3.54(dd);3.63(t); 4.07(t)	CH(2);CH(4,6);CH(1,3); CH(5)	M, CC
38	Methanol	3.37(s)	CH_3	St, M, CC
39	α-Glucose	3.42(t);3.54(dd);3.71(t); 3.74(m);3.84(m);5.24(d)	CH(4);CH(2);CH(3); CH(6);CH(5&6); CH(1);	St, CC
40	Glycine	3.56(s)	CH_2	St, M, CC
41	Glycerol	3.57(m);3.62(m);3.79(m)	CH_2; CH'_2; CH	St, M
42	Glycogen	3.63(m);3.83(m);3.96(m);	CH(3,5,29);CH(6,23,41); CH(4,9); CH(2,17,25)	CC
43	Guanidoacetate	3.80(s)	CH2	CC
44	Serine	3.83(dd); 3.96(m)	CH; CH_2	St, M, CC

45	Hippurate	3.97(d);7.84(d)	CH2;CH(3,5);CH(4);CH(2,6)	CC
46	Inosine	4.28(dd);4.44(t);6.09(d);8.22(s); 8.34(s)	CH(5); N-CH=N	St, M, CC
47	Adenosine	4.30(dd); 8.26(s); 8.35(s)	CH(5); N-CH=N	M, CC
48	NAD+	4.49(dd);4.55(m);6.04(d); 6.09(d);8.18(s);8.21(m);8.43(s); 8.84(d)	CH(29);CH(4,26);CH(2); CH(28);CH(12);CH(38); CH(7);CH(39);CH(37); CH(35)	St, M, CC
49	Allantoin	5.39(s)	CH	M
50	U-Glc	5.61(dd);5.98(m);7.96(d)	CH(2);CH(21,31);CH(32)	M
51	Uracil	5.80(d); 7.53(d)	CH(5); CH(6)	St, M, CC
52	Cytidine	5.89(d);6.06(d);7.83(d)	CH(10); CH(2); CH(11)	CC
53	Uridine	5.90(d); 7.87(d)	CH(10); CH(11)	St, M
54	Fumarate	6.52(s)	CH	St, M, CC
55	Tyrosine	6.89(d); 7.19(d)	m-CH; o-CH	St, M, CC
56	Histidine	7.08(s);7.83(s)	β-CH;CH(5);CH(2);	St, M, CC
57	3-Methylhistidine	7.04(s); 7.68(s)	CH(2); CH(4)	St, M, CC
58	Xanthine	7.93(s)	CH	St, M, CC
59	Nicotinamide	8.24(dd);8.72(dd); 8.94(s)	CH(4);CH(6);CH(2)	St, M, CC

Notes: s: singlet, d: doublet, t: triplet, q: quartet, m: multiplet, dd: doublet of doublet. St: stomach, M: medulla, CC: cerebral cortex. LDL: Low density lipoprotein, VLDL: Very low density lipoprotein, OIV: 2-Oxoisovalerate, GABA: γ-Aminobutyrate, NAA: N-acetyl aspartate, DMG: N,N-Dimethylglycine, GPC: Glycerophosphocholine, NAD+: Nicotinamine adenine dinucleotide, U-Glc: Uridine diphosphate glucose.

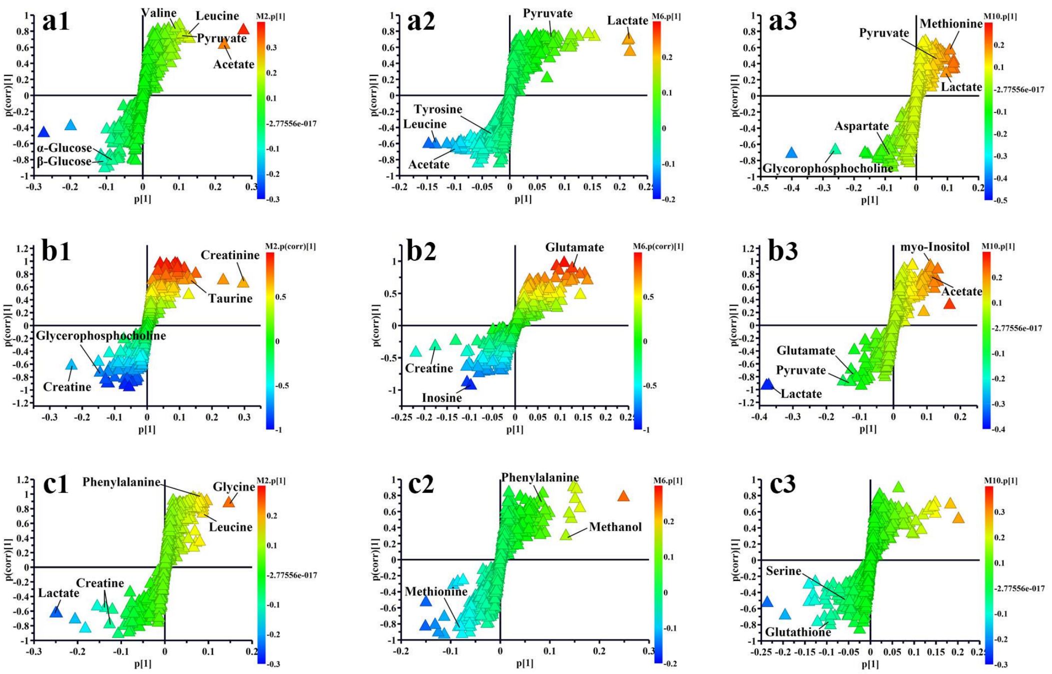

Figure S2 Corresponding S-plots from the GML rats and the control rats in stomach (a1, a2 and a3), cerebral cortex (b1, b2 and b3) and medulla (c1, c2 and c3) tissues for three time points.

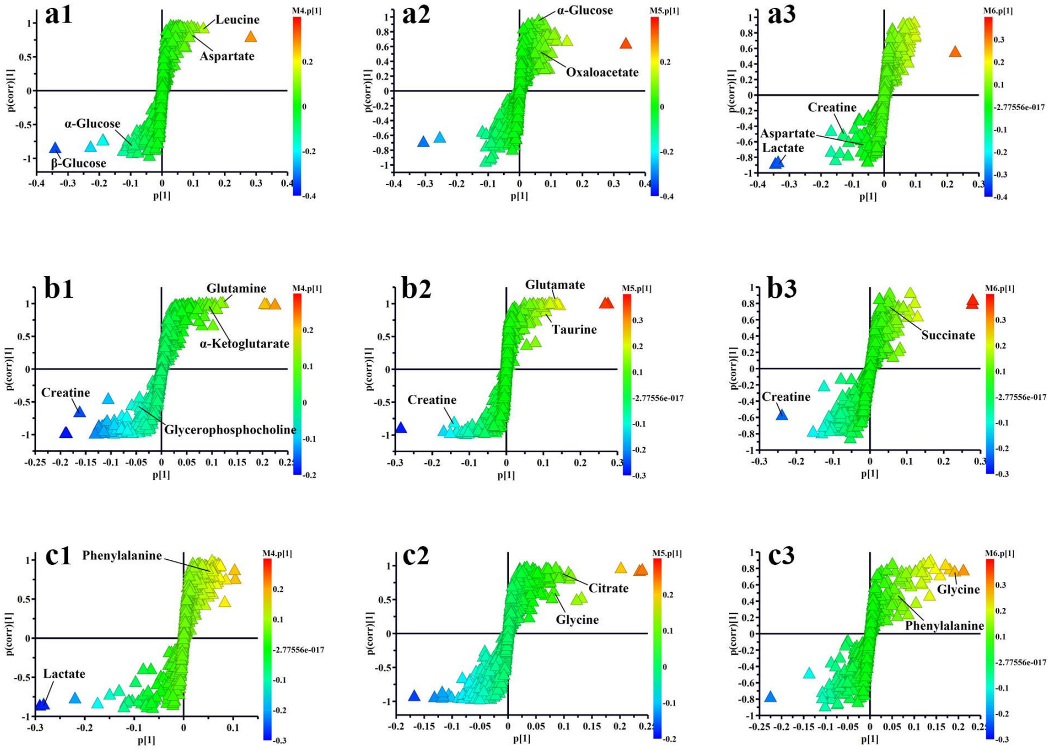

Figure S3 Corresponding S-plots from the GML rats and the GML rats with moxibustion acupoints（MA） treatment in stomach (a1, a2 and a3), cerebral cortex (b1, b2 and b3) and medulla

(c1, c2 and c3) tissues at three time points.

Table S2 The alteration metabolites from stomach tissue

Metabolites	Time point	Relative Concentration[a]			
		Control	GML[b]	MA[c]	MNA[c]
β-Glucose	1 day	0.36±0.07	1.17±0.10	0.52±0.13	0.60±0.23
	4 days	0.92±0.17	0.21±0.10	0.75±0.10	0.93±0.20
	7 days	2.08±0.38	1.74±0.22	2.12±0.10	1.95±0.13
α-Glucose	1 day	0.74±0.08	1.32±0.10	0.97±0.10	1.19±0.11
	4 days	5.72±0.53	3.18±0.55	5.62±0.59	5.14±0.61
	7 days	1.00±0.19	0.83±0.11	1.03±0.05	0.99±0.06
Acetate	1 day	3.21±0.38	2.03±0.08	3.08±0.14	3.20±0.35
	4 days	3.76±0.35	7.18±1.19	4.96±0.46	5.38±0.24
	7 days	2.33±0.14	2.25±0.13	2.99±0.13	3.00±0.07
Pyruvate	1 day	4.72±0.25	3.69±0.13	4.31±0.12	4.09±0.43
	4 days	5.58±0.39	4.03±0.28	5.12±0.49	5.72±0.28
	7 days	5.98±0.39	5.12±0.26	5.07±0.32	4.94±0.16
Oxaloacetate	1 day	2.49±0.14	2.01±0.12	2.38±0.04	2.15±0.22
	4 days	2.96±0.22	2.14±0.11	2.82±0.27	2.98±0.13
	7 days	0.55±0.09	0.43±0.05	0.56±0.04	0.57±0.05
Citrate	1 day	0.49±0.09	0.39±0.07	2.55±0.40	1.09±0.41
	4 days	1.20±0.07	1.04±0.07	1.60±0.15	1.85±0.11
	7 days	1.37±0.16	2.61±0.49	1.52±0.07	1.94±0.09
Isoleucine	1 day	3.30±0.11	2.70±0.19	3.81±0.16	4.02±0.19
	4 days	4.50±0.36	7.55±1.20	6.01±0.51	5.40±0.36
	7 days	1.37±0.17	1.43±0.18	1.95±0.09	1.92±0.09
Leucine	1 day	9.10±0.26	7.55±0.44	11.98±0.44	11.73±0.65
	4 days	13.29±0.95	21.78±3.42	20.42±1.53	21.24±1.18
	7 days	10.10±1.06	10.26±1.18	11.00±0.60	11.56±0.78

**Valine**	**1 day**	**5.61±0.15**	**4.65±0.28**	**7.24±0.20**	**7.14±0.37**
	**4 days**	**8.14±0.65**	**13.19±2.01**	**12.13±0.85**	**12.40±0.54**
	**7 days**	**5.84±0.60**	**6.29±0.71**	**6.70±0.30**	**7.04±0.50**
**Lysine**	**1 day**	**2.28±0.06**	**1.95±0.10**	**3.23±0.17**	**3.07±0.19**
	**4 days**	**3.42±0.35**	**6.77±1.42**	**5.63±0.39**	**5.73±0.13**
	**7 days**	**2.23±0.22**	**2.40±0.25**	**2.89±0.11**	**3.00±0.11**
**Glutamate**	**1 day**	**6.14±0.40**	**4.67±0.14**	**5.58±0.13**	**5.60±0.53**
	**4 days**	**4.88±0.44**	**3.77±0.13**	**4.71±0.40**	**5.12±0.26**
	**7 days**	**2.07±0.15**	**1.77±0.12**	**1.84±0.07**	**1.79±0.05**
**Glutamine**	**1 day**	**5.01±0.26**	**4.13±0.21**	**6.26±0.09**	**5.99±0.32**
	**4 days**	**6.59±0.59**	**6.53±0.40**	**5.81±0.51**	**6.01±0.08**
	**7 days**	**5.21±0.33**	**4.32±0.31**	**4.79±0.17**	**4.71±0.08**
**Tyrosine**	**1 day**	**1.34±0.03**	**1.05±0.06**	**1.68±0.06**	**1.82±0.09**
	**4 days**	**1.57±0.12**	**2.74±0.51**	**2.04±0.20**	**2.13±0.13**
	**7 days**	**1.07±0.10**	**1.11±0.09**	**1.49±0.10**	**1.46±0.04**
**Phenylalanine**	**1 day**	**1.57±0.04**	**1.17±0.09**	**2.05±0.08**	**2.10±0.09**
	**4 days**	**2.06±0.16**	**3.57±0.59**	**3.26±0.29**	**3.22±0.22**
	**7 days**	**2.75±0.13**	**2.83±0.09**	**3.51±0.05**	**3.39±0.09**
**Aspartate**	**1 day**	**0.71±0.02**	**0.62±0.06**	**0.88±0.04**	**0.93±0.02**
	**4 days**	**1.56±0.12**	**1.26±0.15**	**2.59±0.20**	**3.09±0.11**
	**7 days**	**4.48±0.15**	**5.75±0.32**	**6.75±0.28**	**6.48±0.13**

Notes: [a]The relative concentration values are mean ± standard error of the mean (SEM); [b]Red font denote significant increase whereas the blue font indicate significant decrease and the black font denote no significant changes in GML rats compared to the controls; [c]Red font denote significant increase whereas the blue font indicate significant decrease and the black font denote no significant changes in GML rats with moxibustion treatment compared with GML rats.

Table S3 The alteration metabolites from cerebral cortex tissue

Metabolites	Time point	Relative Concentration[a]			
		Control	GML[b]	MA[c]	MNA[c]
Succinate	1 day	1.69±0.04	1.50±0.02	1.56±0.02	1.47±0.01
	4 days	1.57±0.08	1.48±0.04	1.57±0.09	1.60±0.19
	7 days	1.72±0.07	1.62±0.09	1.94±0.08	2.03±0.12
α-ketoglutarate	1 day	0.86±0.04	0.65±0.02	1.76±0.01	1.80±0.02
	4 days	0.55±0.07	0.48±0.04	1.40±0.30	1.23±0.24
	7 days	0.77±0.28	0.96±0.36	1.09±0.38	1.00±0.56
Creatine	1 day	16.36±0.83	20.64±1.48	15.21±1.19	18.32±0.56
	4 days	25.06±2.19	27.56±0.56	19.38±1.76	24.02±0.59
	7 days	27.13±1.29	29.14±2.25	23.84±0.62	23.99±0.98
Glycerophosph-ocholine	1 day	4.76±0.09	5.31±0.15	4.89±0.10	5.05±0.10
	4 days	6.12±0.14	6.30±0.17	4.36±0.11	4.93±0.09
	7 days	5.00±0.12	4.66±0.09	4.63±0.17	4.85±0.16
Fumarate	1 day	1.33±0.07	0.87±0.03	1.00±0.03	0.92±0.02
	4 days	0.69±0.10	0.66±0.09	1.06±0.22	1.13±0.26
	7 days	0.47±0.14	0.45±0.18	0.47±0.15	0.48±0.18
Taurine	1 day	9.97±0.28	8.75±0.25	10.00±0.16	9.37±0.09
	4 days	7.08±0.65	6.80±0.68	11.29±0.43	9.02±0.74
	7 days	9.49±0.67	9.04±0.37	9.38±0.45	8.40±0.80
Glutamate	1 day	9.50±0.08	8.97±0.11	10.22±0.05	9.94±0.03
	4 days	6.84±0.06	6.40±0.06	12.71±0.38	11.47±0.26
	7 days	10.47±0.17	11.55±0.22	10.84±0.18	11.74±0.40
Glutamine	1 day	1.36±0.03	0.92±0.03	2.97±0.04	2.92±0.03
	4 days	3.22±0.10	2.75±0.05	6.67±0.50	5.51±0.40
	7 days	9.11±0.24	9.85±0.19	9.46±0.19	10.27±0.22

Notes: [a]The relative concentration values are mean ± standard error of the mean (SEM); [b]Red font

denote significant increase whereas the blue ones indicate significant decrease and the black ones denote no significant changes in the GML rats compared with the controls; [c]Red font denote significant increase whereas the blue ones indicate significant decrease and the black ones denote no significant changes in GML rats with moxibustion treatment compared with GML rats.

Table S4 The alteration metabolites from medulla tissue

Metabolites	Time point	Relative Concentration[a]			
		Control	GML[b]	MA[c]	MNA[c]
Lactate	1 day	54.94±2.57	65.48±3.23	45.61±1.65	43.91±3.35
	4 days	40.34±0.88	41.62±0.98	45.48±2.03	45.85±1.91
	7 days	44.46±1.72	47.05±2.05	45.96±2.36	47.88±1.61
Citrate	1 day	2.06±0.05	1.01±0.17	1.75±0.12	1.93±0.07
	4 days	6.12±0.09	5.95±0.16	7.12±0.10	7.69±0.11
	7 days	5.62±0.12	5.52±0.11	5.73±0.08	5.71±0.12
Methionine	1 day	3.55±0.13	2.71±0.08	3.46±0.12	3.50±0.13
	4 days	1.91±0.06	2.16±0.05	2.14±0.07	1.91±0.15
	7 days	2.64±0.04	2.89±0.10	2.68±0.09	2.81±0.04
Glycine	1 day	7.49±0.37	4.90±0.28	6.08±0.24	6.72±0.12
	4 days	17.54±0.50	15.82±0.50	17.18±0.21	19.38±0.56
	7 days	8.02±0.42	8.92±0.27	9.23±0.22	9.04±0.42
Serine	1 day	2.28±0.05	1.78±0.05	2.28±0.15	2.29±0.14
	4 days	1.44±0.05	1.66±0.03	1.87±0.05	1.54±0.15
	7 days	1.73±0.02	1.86±0.05	1.80±0.08	1.79±0.05
Glutathione	1 day	0.54±0.02	0.23±0.06	0.73±0.14	0.64±0.12
	4 days	0.57±0.04	0.58±0.02	0.72±0.02	0.73±0.05
	7 days	0.50±0.02	0.60±0.03	0.63±0.03	0.60±0.03
Phenylalanine	1 day	1.80±0.09	1.05±0.09	1.80±0.15	1.81±0.09

**4 days**	**4.04±0.09**	**3.73±0.04**	**4.10±0.09**	**5.12±0.37**
**7 days**	**8.31±0.19**	**8.74±0.18**	**9.22±0.11**	**9.07±0.25**

Notes: [a]The relative concentration values are mean ± standard error of the mean (SEM); [b]Red font denote significant increase whereas the blue ones indicate significant decrease and the black ones denote no significant changes in the GML rats compared with the controls; [c]Red font denote significant increase whereas the blue ones indicate significant decrease and the black ones denote no significant changes in GML rats with moxibustion treatment compared with GML rats.

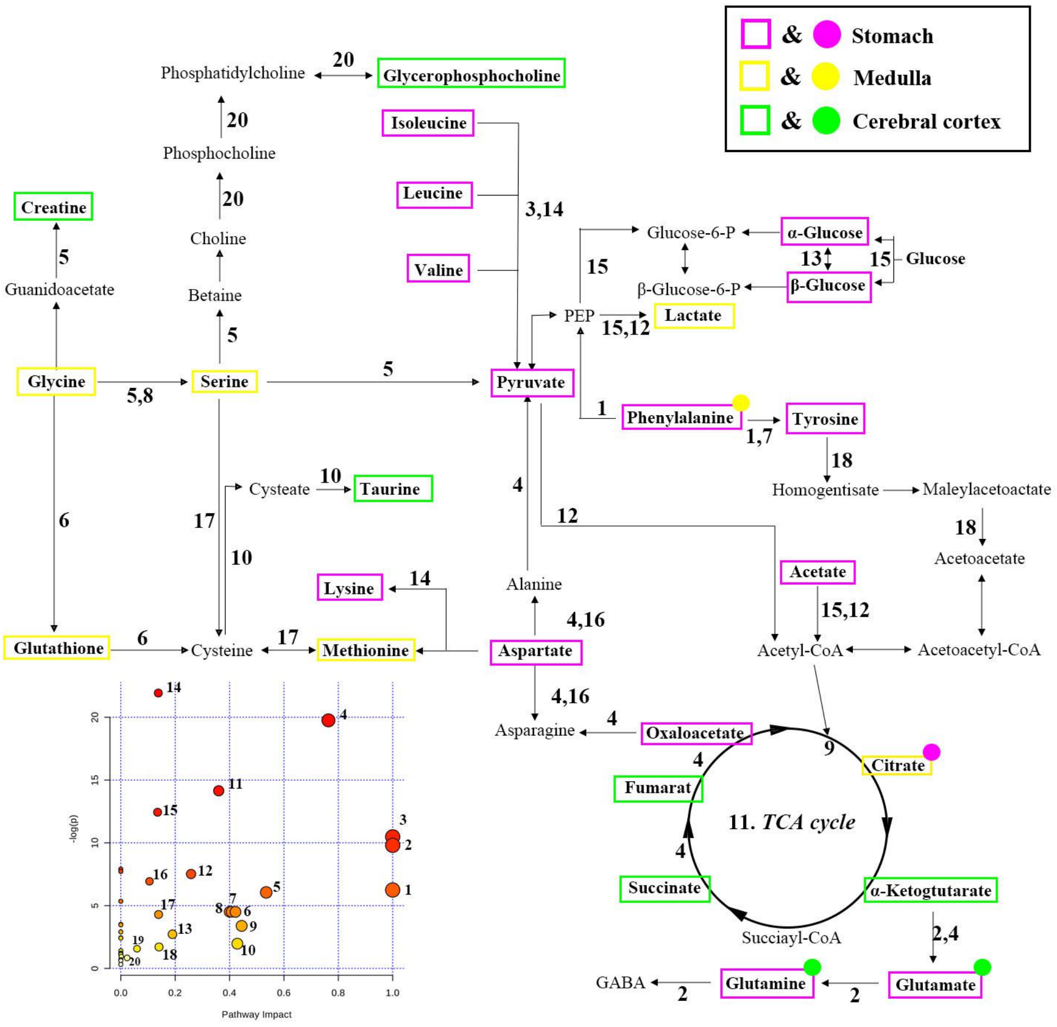

Figure S4 Summaries of metabolic pathways in gut-brain integration altered moxibustion treatment on GML rats. Moxibustion intervention showed beneficial effects by regulating many

GML-induced metabolic changes involved in energy metabolism, amino acids metabolism and membrane metabolism. (1, Phenylalanine, tyrosine and tryptophan biosynthesis; 2, D-Glutamine and D-glutamate metabolism;3, Valine, leucine and isoleucine biosynthesis; 4, Alanine, aspartate and glutamate metabolism; 5, Glycine, serine and threonine metabolism; 6, Glutathione metabolism; 7, Phenylalanine metabolism; 8, Methane metabolism; 9, Glyoxylate and dicarboxylate metabolism; 10, Taurine and hypotaurine metabolism; 11, Citrate cycle (TCA cycle); 12, Pyruvate metabolism; 13, Starch and sucrose metabolism; 14, Aminoacy-tRNA biosynthesis; 15, Glycolysis or Gluconeogenesis; 16, Arginine and proline metabolism; 17, Cysteine and methionine metabolism; 18, Tyrosine metabolism; 19, Primary bile and biosynthesis; 20, Glycerophospholipid metabolism.)
